# Hyperkalemia Induced by the Sequential Administration of Metoprolol and Carvedilol

**DOI:** 10.1155/2018/7686373

**Published:** 2018-10-15

**Authors:** S. Serge Barold, Scott Upton

**Affiliations:** ^1^Department of Medicine, University of Rochester School of Medicine and Dentistry, Rochester, NY, USA; ^2^San Diego Internal Medicine and Pediatrics Associates, San Diego, CA, USA

## Abstract

This report describes the occurrence of asymptomatic hyperkalemia induced by the sequential administration of metoprolol and carvedilol in an 81-year-old man with type II diabetes and stable stage III renal insufficiency. The potassium level rose to 5.6–5.7 mEq/L with metoprolol and normalized when the agent was discontinued. However, the potassium level rose again to 5.6 mEq/L after the administration of carvedilol but the level normalized by halving the dose. The observations of hyperkalemia induced by two different *β*-blocker drugs in the same patient confirm that this side effect is common to all *β*-blocker drugs.

## 1. Introduction

Hyperkalemia is a rare side effect of *β*-blocker drugs [1–3]. In this report, we describe the occurrence of metoprolol-induced hyperkalemia in a patient with type 2 diabetes and stage III renal dysfunction. The potassium level normalized after metoprolol was discontinued, but the administration of carvedilol subsequently induced hyperkalemia. The development of hyperkalemia in the same patient induced by two different *β*-blocker drugs has not been previously reported.

## 2. Case Report

An 81-year-old man presented with asymptomatic of hyperkalemia (5.7 mEq/L). The past history included mitral valve repair and coronary bypass surgery 27 years previously, mild type II diabetes for 12 years, renal insufficiency (stage III) with a stable creatinine level of 1.5 mg/dL for 8 years and prostatic hypertrophy. An echocardiogram performed 6 months previously revealed normal left ventricular function and minimal mitral regurgitation and an ECG documented sinus rhythm and complete left bundle branch block. Medications included Avodart 0.5 mg qd, atorvastatin 80 mg qd, aspirin 325 mg qd, Januvia 25 mg qd, and metoprolol tartrate 50 mg bid. The latter had been prescribed 6 years previously for frequent symptomatic atrial premature beats with an excellent clinical result so that metoprolol at the same dose (all tartrate preparation) was continued up to the most recent evaluation. The potassium level had always been normal before the administration of metoprolol. Afterwards, routine testing with serum electrolytes every 6 or 12 months consistently revealed a potassium level of 5.4 mEq/L. Then, for the last 2 years regular routine testing revealed a potassium level fluctuating between 5.6 and 5.7 mEq/L. Finally, metoprolol was discontinued and 18 days later the potassium level normalized at 4.2 mEq/L. Carvedilol was started and then increased to 12.5 mg bid. After about 10 days on this dose, the potassium level increased to 5.6 mEq/L. The dose of carvedilol was therefore reduced to 6.25 mg bid. A follow-up potassium level 2 weeks after the start of the lower carvedilol dose was 5.0 mEq/L which is at the upper limit of normal for the testing laboratory.

## 3. Discussion

*β*-Blocker-induced hyperkalemia is a rare nonspecific side effect of *β*-blocker therapy as illustrated by the effect from two different *β*-blockers in the same patient. It appears from a review of the literature that a biological difference between the 2 available forms of metoprolol, tartrate or succinate, is unlikely.

### 3.1. Metoprolol

A 2018 FDA report evaluated the incidence of hyperkalemia (level not stated) in 24,296 patients taking metoprolol succinate and found 287 patients with hyperkalemia [4]. The patients with hyperkalemia were taking ramipil (41.5%) or spironolactone (10.4%). No other potassium-retaining drugs were taken. An increased creatinine was present in 20.56% and acute renal failure in 33.80% of the patients. The FDA report suggests that metoprolol-induced hyperkalemia occurs in about 0.5% of the patients without acute renal failure and can occur in patients without diabetes or renal dysfunction. Yet, there are only two Medline citations about this side effect of metoprolol. One case involved a 45-year-old hypertensive diabetic man with advanced renal failure on hemodialysis who developed marked recurrent hyperkalemia while on metoprolol therapy (full preparation not stated) [1]. The other case documented hyperkalemia as a result of a suicide with metoprolol [5].

### 3.2. Propranolol

Propranolol has rarely been reported to induce hyperkalemia in the adult patient, though it is well documented as a serious problem when it is used to treat hemangiomas in infants [3, 6, 7].

### 3.3. Carvedilol

In our case, the potassium level rose to 5.6 mEq/L when the patient (with type III renal insufficiency) was on carvedilol 12.5 mg bid but it normalized (though at the upper limit of normal—5 mEq/L) when the dose of carvedilol was reduced to 6.25 mg bid. A similar observation was recently reported about carvedilol-induced hyperkalemia in a 69-year-old man with hypertension, type II diabetes, and stage III renal insufficiency who was hospitalized for abdominal pain [2]. He had been on carvedilol 3.125 mg bid and lisinopril 40 mg qd. The serum potassium was 4.8 mEq/L. When carvedilol was increased to 6.25 mg bid, the serum potassium rose to 6.7 mEq/L without any change in lisinopril dosage or administration of other potassium-retaining drugs. A simple reduction back to the original dose normalized the potassium level [2]. These two reports suggest that the development of hyperkalemia is dose dependent.

### 3.4. Labetalol

Intravenous labetalol has been associated with severe hyperkalemia for the treatment of acute hypertension in hemodialysis patients, renal transplant patients, and in preeclampsia [8–10].

### 3.5. Timolol Eyedrops

Severe hyperkalemia occurred in a patient with glaucoma following the use of timolol maleate eyedrops, but the serum potassium level normalized upon discontinuation of the eyedrops [11]. However, hyperkalemia recurred following a rechallenge with the same eyedrops and the potassium level normalized again when the eyedrops were withdrawn.

## 4. Mechanisms

It is well known that adrenergic agents decrease serum potassium and hence are used to treat life-threatening hyperkalemia. *β*2 adrenergic agonists drive potassium into the cells by increasing the activity of the Na-K pump. Thus, a catecholamine surge tends to lower the serum potassium [12]. They also activate the inwardly directed Na-K-Cl cotransporter, a protein that aids in the active transport of sodium, potassium, and chloride into cells [12]. Yet, the opposite effect with the production of hyperkalemia by the inhibition of the sympathetic system as with *β*-blockers has only occasionally been reported. The propensity to hyperkalemia may be masked or unrecognized because *β*-blockers are commonly administered together with drugs that affect potassium balance.

Barring the acute administration of a *β*-blocker drug, there are only 4 well-documented case reports of *β*-blocker-induced hyperkalemia from sustained therapy (3 from the literature and one described herein). Three cases had associated renal dysfunction and diabetes, while one had none of these associations. Insulin similar to epinephrine enhances the activity of the Na-K pump so that an anti-insulin effect in diabetes would favor the movement of potassium outside the cells [12]. Renal insufficiency decreases the cell membrane potential and thus leads to translocation of potassium to the extracellular fluid [13]. The above clinical cases confirm the FDA data that renal insufficiency is a strong predisposing factor to the development of *β*-blocker-induced hyperkalemia. These cases also suggest a probable association with diabetes, though the FDA data revealed a 10% incidence perhaps because of a different patient population. Finally, it is possible that hyperkalemia may be due to a genetic predisposition.

## 5. Conclusion

We have reported metoprolol-induced and carvedilol-induced hyperkalemia. Such hyperkalemia is rare and seems to be a common side effect of *β*-blockers. Hyperkalemia is more common in patients with renal insufficiency and probably in those with diabetes. Identification of this form of hyperkalemia may avoid unnecessary investigations and prevent untoward effects. In patients with stable renal insufficiency, *β*-blocker-induced hyperkalemia should not be attributed to progression of kidney disease causing unwarranted alarm and inappropriate therapy. This form of hyperkalemia may be dose dependent so that normalization of the potassium level may not necessarily require complete withdrawal of therapy.
